# Dynamic Resource Allocation in Hybrid Access Femtocell Network

**DOI:** 10.1155/2014/539720

**Published:** 2014-03-20

**Authors:** Afaz Uddin Ahmed, Mohammad Tariqul Islam, Mahamod Ismail, Mohammad Ghanbarisabagh

**Affiliations:** ^1^Department of Electrical, Electronic and Systems Engineering, Universiti Kebangsaan Malaysia, 43600 Bangi, Selangor, Malaysia; ^2^Department of Electrical Engineering, Universiti Malaya, 50603 Kuala Lumpur, Malaysia

## Abstract

Intercell interference is one of the most challenging issues in femtocell deployment under the coverage of existing macrocell. Allocation of resources between femtocell and macrocell is essential to counter the effects of interference in dense femtocell networks. Advances in resource management strategies have improved the control mechanism for interference reduction at lower node density, but most of them are ineffective at higher node density. In this paper, a dynamic resource allocation management algorithm (DRAMA) for spectrum shared hybrid access OFDMA femtocell network is proposed. To reduce the macro-femto-tier interference and to improve the quality of service, the proposed algorithm features a dynamic resource allocation scheme by controlling them both centrally and locally. The proposed scheme focuses on Femtocell Access Point (FAP) owners' satisfaction and allows maximum utilization of available resources based on congestion in the network. A simulation environment is developed to study the quantitative performance of DRAMA in hybrid access-control femtocell network and compare it to closed and open access mechanisms. The performance analysis shows that higher number of random users gets connected to the FAP without compromising FAP owners' satisfaction allowing the macrocell to offload a large number of users in a dense heterogeneous network.

## 1. Introduction

Cellular operators these days aim to provide higher number of multimedia contents to attract the new generation customers to increase their revenue. Voice services are mostly characterized by a subscriber's number, while the data service is characterized by the use of a vast number of applications and protocols. As the data traffic is increasing exponentially, a need for proper resource management has risen for better quality of service (QoS). Deployment of FAP is primarily supported by the argument of improved indoor coverage for consumers and substantial cost savings for operators due to capacity offload. It is an effective alternative to divert and carry out a big portion of the traffic from the macrocell/macrobase station (MBS). The key issue that restricts the vast implementation of FAP is the lack of effective schemes to mitigate interferences. FAP causes potential interferences with colocated MBS and neighboring FAPs operating in the same frequency. Selection of access-control mechanisms in femtocell has a deteriorating effect on network performance. The operation of access-controlled femtocell greatly depends on the mechanism whose sole purpose is to decide whether the user can connect to the cell [1, 2].

**Figure 1:**
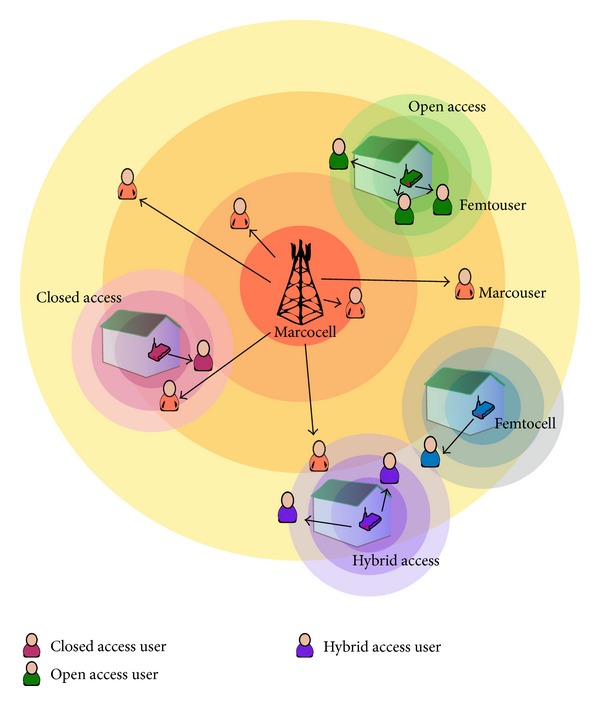
Different access control in mechanism femtocell network.

Open access, close access, and hybrid access are the three existing access-control methods that decide users' connectivity to the FAP. In open access, whenever the users are within the range of a FAP, they get connected to the FAP easily. This includes a new set of signalling congestion in the network, as the number of handover attempts gets higher compromising the level of sharing and security concerns for the regular user. In the case of closed access, only particular users get access to the FAP, thus avoiding unwanted traffic congestion and possible interferences. In this case, the QoS is guaranteed at the expense of decreasing spectral efficiency. Hybrid access transacts with both challenges by tuning the resource ratio according to the number of femtocell owners and subscribers. A limited amount of resources is available to the users who are within the coverage range and a “closed subscriber group” possesses the privilege to use the maximum service. Hybrid access reaches a compromise between the impact on the performance of subscribers and level of access granted to nonsubscribers, allowing them to possess a limited amount of features [3, 4].  Figure 1 shows different access control mechanism in a femtocell network.

From the operators' point of view, open access is suitable for interferences reduction and cost optimization. However, serving to an unknown number of guests may degrade the QoS of the FAP owners. On the contrary, FAPs are especially designed for indoor coverage to ensure the maximum service for their owners. Recent studies have focused on the performance of closed access, open access, and resource-allocation management in heterogeneous network [5–18]. In [19] a resource-allocation technique in OFDMA femtocell network was considered with instantaneous FAPs power control mechanism for a target SINR. Joint resource-allocation and admission control design for dynamic frequency sharing was discussed in [20, 21]. A “3-ON-3 Femtocell Clustering Architecture” concept was developed in [22], where 3 FAPs form a cluster unit that transmits 3 power levels to serve 3 different types of user based on their priority level. In [23] both the centralized and decentralized approaches had been considered, but the cochannel deployment is avoided which leads to less spectrum efficiency from the frequency reuse prospective, thus placing this approach under dispute. Finally, authors in [24] considered a joined centralized-distributed approach for resource allocation in an open access cochannel femtocell network where the level of users was not distinguished.

Cochannel deployment is preferred by the operators as it contains low cost and better spectral efficiency. Although in shared-bandwidth approaches, majority of which are currently being developed and deployed, the effect of interference is a big concern. Most of the techniques that deal with resource allocation of the frequency and energy at the same time are limited to single-tier networks. For multitier networks, the resource allocation should be more developed to handle the for multitier networks, the recource allocation should be more developed to handle the associate challenges. Resource allocation that is based centrally incurs excessive data transmission. Moreover, the ad hoc nature of femtocell makes the centralized control quite infeasible. However, the local approach provides anatomy in the system that speeds up the performance but suffers from quality degradation due to lack of essential data regarding interferences and resources allocated in the neighboring cells. In this paper, a joined centralized-distributed technique is recommended to design a dynamic resource-allocation scheme for cochannel hybrid access-control femtocell network [25]. The system switches intelligently between the two modes based on the FAP owners' required throughput and users' density in a particular area of the network. It is a combined technique to utilize the unoccupied resources to maximize the QoS for the floating users and offloading MBS during overload condition. The rest of the paper is organized as follows: system analysis and modeling in Section 2, DRAMA-based hybrid access in Section 3, simulation results in Section 4, and conclusion in Section 5.

## 2. System Analysis and Modeling

**Figure 2:**
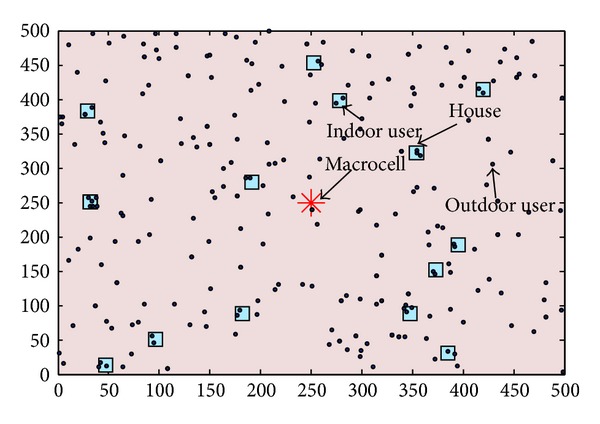
Sample layout of the simulation.

A spectrum sharing two-tier heterogeneous network is considered with OFDMA in the downlink. An area of 500 m × 500 m is studied that contains one MBS in the middle and variable number of FAPs distributed within the area using homogeneous Poisson Point Distribution (PPP). All the FAPs are in the middle of a rectangular house. The FAPs do not overlap with each other or with the MBS. During the selection, all the users are 1 m apart from the MBS and FAPs. For outdoor users, 3GPP-LTE standard HATA small/large city model is used as it is more realistic for dense urban environment. WINNER II channel models are used for indoor path-loss scenarios for both line-of-sight (LOS) and non- line-of-sight (NLOS) users [26]. Wall penetration losses are also considered for the users who are in NLOS. To simulate the performance of the all the access-control mechanisms, a simulation environment is developed in MATLAB. A sample of the simulation layout is given in Figure 2.

OFDMA in LTE is robust against multipath interference and frequency selectivity [27]. In OFDMA network, FAPs have the advantage of allowing the allocation of orthogonal frequency/time resources to users. In the simulation, the whole bandwidth is divided into certain resource blocks (RBs) and certain numbers of subcarriers are allotted to each of the RB. All randomly deployed FAPs operate in the same bandwidth. The RB assumption is adopted from the 3GPP-LTE standard concept defined in [28]. A narrow channel appears relatively flat in the frequency domain and the process of equalization is thus simplified. Therefore, channel capacity is relatively higher with narrow bandwidths and it does not change by dividing the frequency band into narrower frequency bins.

For *x* number of subcarriers, the signal-to-interference-plus-noise-ratio (SINR) expression for MBS user is

(1) $$\begin{array}{c} \text{SIN}{\text{R}}_{m,x}=\frac{P_{M,x}G_{M,m,x}D_{M}^{-\alpha_{m}}}{N_{0}\Delta f+\sum_{i\in F_{i}}\phi P_{i,x}G_{i,m,x}D_{i}^{-\alpha_{mi}}}, \end{array}$$

where *N*_0_, Δ*f*, *P*_*M*,*x*_, *G*_*M*,*m*,*x*_, *D*_*M*_, *P*_*i*,*x*_, *G*_*i*,*m*,*x*_, and *D*_*i*_ are the white noise power spectrum density, subcarrier spacing, transmission power from MBS, random channel gain from MBS to MBS user, transmitting power of interfering FAPs, random channel gain from FAP to MBS user, and distance from FAP to MBS user, respectively. *α*_*m*_ and *α*_*mi*_ are the path-loss exponents of the link from MBS and from FAP to the user, respectively. *F*_*i*_ is the set of interfering FAP for that particular MBS user. *ϕ* is the penetration loss for a single wall.

The SINR expression for the FAP user is

(2) SINRf,k =PF,kGF,f,kDF−αfN0Δf+ϕPM,kGM,f,kDM−αmf+∑i∈Fiϕ2Pi,kGi,f,kDi−αfi,

where *P*_*F*,*k*_, *G*_*F*,*f*,*k*_, *D*_*F*_, *G*_*M*,*f*,*k*_, and *G*_*i*,*f*,*k*_ are the transmission power from FAP to FAP user, random channel gain for FAP user, distance from FAP to user, random channel gain from MBS to FAP user, and random channel gain from interfering FAP to targeted FAP user, respectively. *α*_*f*_, *α*_*mf*_, and *α*_*mf*_ are the path-loss exponent of the link from FAP, MBS, and interfering FAP to the targeted user, respectively.

## 3. Dynamic Resource-Allocation Management Algorithm- (DRAMA-) Based Hybrid Access

FAP includes not just the base station itself but also the controller that enables local radio resource control. This connects back to the mobile operator core at a higher point for central authentication and management, which addresses the scalability concerns above, as the resource is located locally. Within the coverage range of FAP, users give the highest priority to the FAP for better signal quality. MBS wants to transfer “excess load” towards FAP to reduce user congestion and signaling overhead. Whenya FAP does not allow access; the user tries to stay connected to the MBS to avoid call drop. In hybrid access, FAP allows random users to get a better QoS. Based on the operator's network planning, the service for random users varies. In most of the cases, FAP assigns a ratio of the total resources to the guest users. This degrades owners' satisfactory level of service. In this scheme, the FAP owners are allowed to select the minimum level of uninterrupted service they want to enjoy. Initially, FAP ensures uninterrupted service to the owners. Subsequently it utilizes the rest to serve a particular number of random users. Depending on the user congestion in the network, radio network controller (RNC) selects a minimum level of service for each FAP to assign to the random users. The trick is that whenever the network has less congestion of users, the FAP serves a fewer number of users, allocating more resources to each of them. However, for higher user congestion, FAP serves more users with fewer resources. The resource allocation must be high enough to cross the required threshold level for handover. The network balances the total traffic ensuring the maximum level of resource efficiency. Bonus usage or reward tariff should be awarded to the owners for offloading a certain percentage of the total load to encourage the sharing of idle resources. Also depending on the pricing policy of the network operators, special tariffs at home can be applied for calls placed under femtocell coverage. As the proposed scheme consists of both centralized and distributed resource management approaches, the radio network controller (RNC) controls the network centrally and FAP controls the rest locally. FAPs connect to the RNC through the backhaul connection. The central controller determines the relative position of the FAPs and MBS. An X2 connection measures and conveys the downlink interference coordination between MBS and FAPs. The DL-HII (Downlink-High Interference Indicator) generates the necessary signal and share through the wired backbone [29]. RNC defines a functioning area for each MBS. Number of active FAPs and users for a constant period of time determines the execution of the scheme for the functioning area.

The network divides all the users in three categories, FAP owners, random users who are under FAP service, and MBS users. Customarily the FAP owners are always within the coverage of their own FAP; otherwise, they are treated as the floating users. Corresponding FAPs allocate adequate resources to the owner(s) so that, overcoming all the losses, FAPs ensure the minimum throughput asked by the owners' (the minimum throughput is shown in Table 2). The rest of the resources are distributed to the random users under the FAPs coverage. The selection of random users is based on the level of service which is subjected to cross-tier interference due to poor MBS coverage and strong FAP interferences. Network allocates resources based on total population of the functioning area. The allocated resources are high enough to insist the MBS users to get handed-over to the nearest FAP. After a predefined interval, the network updates the status of the active FAPs and users and reassigns the resources according to the scheme. The RNC executes certain tasks and the rest will be carried out by the FAPs, avoiding unnecessary delay in assessment. RNC ensures the mobility management undertaking the owners and random user access in the FAPs besides doing its regular function, like, link management, call processing, handling FAPs and MBS, assigning spectrum as RBs, and controlling handover mechanisms. FAPs assign spectrum locally, monitor interference level, get feedback from users in the uplink, and synchronize the strongest signal with the desired macrosignal. In addition, hierarchical cell structure (HCS) can also be introduced to distinguish between MBS and FAP and to execute rules for users of different priority level in each layer [30].

The number of active MBS users in the functioning area and the number of owners in each FAP are *N*_*m*_ and *N*_*f*_, respectively. *N*_*r*_ is the number of MBS users who get access to a FAP service. For users under a FAP's coverage in the network,

(3) $$\begin{array}{c} C_{ff}\propto C_{fr},\;\left\{N_{r}\in ℜ\right\}, \end{array}$$

where *C*_*ff*_ and *C*_*fr*_ are the FAP owners throughput and random users throughput who got access to FAP service, respectively.

Assume a hybrid resource distribution constant *K*_*r*_ as follows:

(4) $$\begin{array}{c} K_{r}=\frac{C_{r}}{C_{f}},\;\left\{x\in K_{r}:0<x<1\right\}. \end{array}$$

If the value of *K*_*r*_ = 1, it will act as an open access FAP and if *K*_*r*_ = 0, it will work as a closed access FAP. The cells will assign spectrum as RBs and for a given time interval the transmission power to all the RBs is equal for both MBS and FAPs. Now the throughput of a particular MBS user which gets handed over to FAP can be expressed as

(5) $$\begin{array}{c} C_{fr}=\frac{\delta_{\text{FAP}}}{N_{f}+N_{r}},\\ \delta_{\text{FAP}}=N_{\text{RB}}C_{\text{RB}}\Delta f\text{lo}{\text{g}}_{2}\left(1+\alpha \text{SIN}{\text{R}}_{f,r}\right),\\ C_{mr}=\frac{\delta_{\text{MBS}}}{N_{m}-\sum_{i=1}^{F-1}N_{r}},\\ \delta_{\text{MBS}}=N_{\text{RB}}C_{\text{RB}}\Delta f\text{lo}{\text{g}}_{2}\left(1+\alpha \text{SIN}{\text{R}}_{m,r}\right), \end{array}$$

where *C*_*mr*_, *C*_*fr*_, *N*_RB_, *C*_RB_, Δ*f*, and *α* are the throughput of random users under MBS service, throughput of random users under FAP service, number of resource blocks, subcarrier per resource block, subcarrier spacing, and *α* is the constant for target bit error rate (BER), respectively.

For maximum random users' throughput and maximum number of MBS offloading, the optimization problem can be stated as

(6) $$\begin{array}{c} \overset{}{\underset{\max}{\sum }}k=\frac{\delta_{\text{FAP}}}{C_{ff}\left(N_{f}+\sum_{\max}N_{r}\right)}\\ \text{Subjected to}\;\;C_{fr}\geq C_{mr},\;N_{r}<N_{m}-\overset{F-1}{\underset{i=1}{\sum }}N_{r}, \end{array}$$

where *F* is the number of active FAPs in the functioning area under the MBS coverage.

If the users provide continuous feedback in the uplink about the SINR for the assigned RB to its FAP or MBS and if the imperfection of proper feedback and channel estimation is not considered, the value of the hybrid constant and number of random users for a FAP are as follows:

(7) $$\begin{array}{c} \overset{}{\underset{\max}{\sum }}k=\frac{\delta_{\text{FAP}}C_{mr}}{C_{mr}\left(N_{f}+N_{m}\right)-\delta_{\text{MBS}}C_{ff}},\\ \overset{}{\underset{\max}{\sum }}N_{r}=\frac{C_{mr}\left(N_{f}-N_{m}-C_{ff}N_{f}\right)-\delta_{\text{MBS}}C_{ff}}{C_{mr}C_{ff}}. \end{array}$$

The FAP will allow *N*_*r*_ number of random users under its coverage to ensure the satisfactory level of service for both fixed and random users. If the assigned bandwidth for the random users is not high enough, the user might experience a lower service even if they get better coverage. So, any random user who gets better service from MBS, will not change serving cell.

## 4. Simulation Result

The simulations are event-based and developed according to 3GPP standards. The plotted values are an average of 1000 independent simulations. The assumed system parameter for the simulation is given in Table 1.

The standard length of the cyclic prefix in LTE is 4.69 *μ*s. This enables the system to tolerate path variations of up to 1.4 km with the symbol length set to 66.7 *μ*s. Each subcarrier can carry maximum data rate of 15 Ksps (kilo-symbols per second). Modulation 64-QAM represents 6 bits per symbol. Therefore, 10 MHz can provide a raw symbol rate of 9 Msps or 54 Mbps. This enables the system to compartmentalize the data across standard numbers of subcarriers [31].

**Figure 3:**
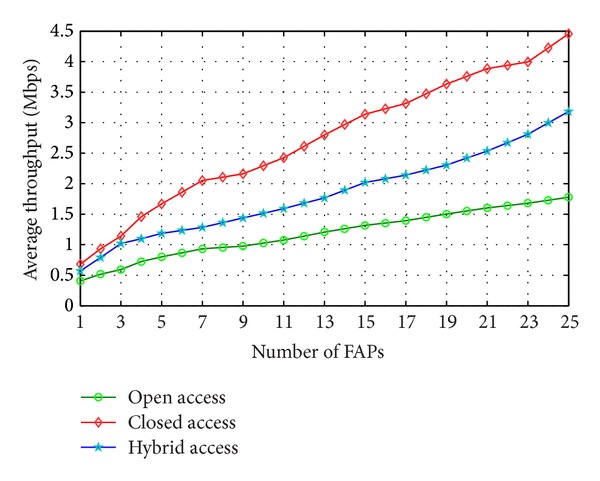
Average throughput of total users for variable numbers of FAPs.

The performance of a resource-allocation scheme is evaluated based on different deployment densities of FAPs within the functioning area. The general performance of open access, closed access, and hybrid access is shown in Figure 3. In case of closed access, the throughput of FAP owners is very high compared to the MBS users that boost up the average throughput of the total users of the network. Hybrid access on the other hand using this scheme shows a better performance than the open access. The average throughput of every access mechanism increases along with the FAPs deployment density.

**Figure 4:**
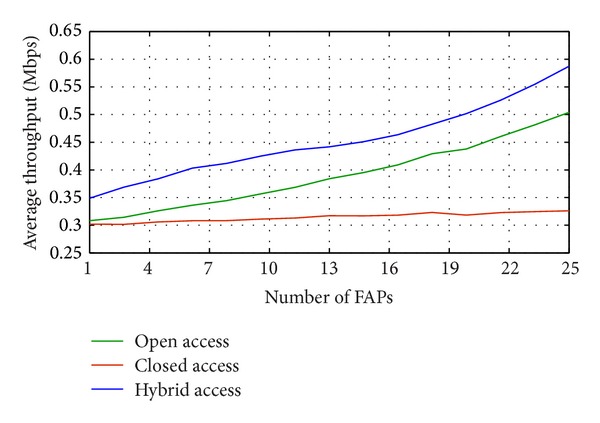
Average throughput of random users for variable number of FAPs.

Figure 4 illustrates the average throughput against variable numbers of deployed FAPs. The performance of hybrid access users is better than that of the other two access mechanisms. Cell edge users who are mostly subjected to the interference get under the FAP's service and FAP is only open for a particular number of random users, as it has to ensure a better service to the existing connected users. In contrast, the open access allows any user who grasps a better signal level under its coverage area. It does not have any priority level settings for the owners and for the random users. Random users always look for a better signal level from the neighboring cells which causes increasing numbers of handover attempts. As a result, the average throughput of the open access is lower than the hybrid access.

**Figure 5:**
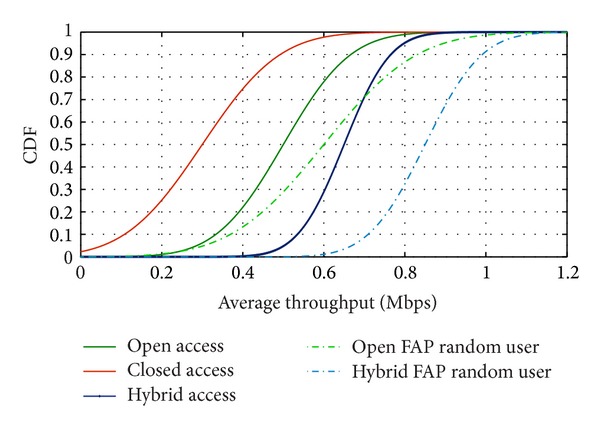
Average throughput of random users for different access mechanisms.

A resulting cumulative function is given in Figure 5. The deviation of average throughput for random users in hybrid access is much less than both open and closed accesses. In the case of open access and hybrid access, users who get access to the FAP service possess comparatively a higher average throughput. The “- -” line shows the average throughput of the random FAP users. FAP only allows a distinct number of users in hybrid access that confirms a higher throughput than the open access. Closed access does not have any mechanism to allow access to the random users. In dence femtocell network, spectrum sharing mandated by the means of co-channel femtocell deployment, has an adverse effect on the system's throughput and the quality of service.

To see the performance of the hybrid access from the users divesting prospective, three sets of owner throughput are considered as the accessibility of the FAPs' unoccupied resources mainly depends on the FAP owners' demand.

**Figure 6:**
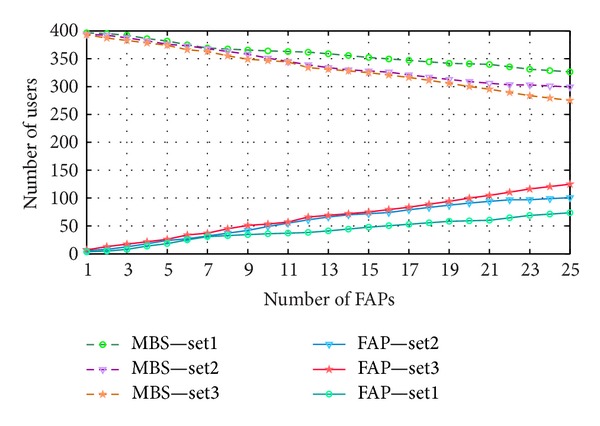
Users served by FAPs and MBS.

Figure 6 shows the number of users occupied by the MBS and FAPs. The owner's minimum throughput levels are set consequently in the system. For set 3, which is the lowest, the numbers of FAP occupied users are the highest. The number of owners per FAP is fixed and an increasing number of random users get access to the FAP with an increasing number of deployment densities. While for set 2 and set 1, the performance of user offloading decreased correspondingly.

Practically in any dense network, the total number of floating users is always higher than the owners. Thus, the escalation of the overall QoS of the operators depends upon random users' consumption. Considering the proposed scheme from the perspective of network performance and subscribers' satisfaction, hybrid access mechanism shows a better performance. The throughput of the FAP owners is above the minimum satisfactory level, while for the random users, it is the maximum. Compared with the open access, the hybrid access confirms 10%–35% better performance for the random users, indicating that the FAP-to-FAP interference is reduced significantly. MBS gets a chance to handover users who are more subjected to cross-tier interference. Because of the reduction of MBS-user congestion, it lessens the chances of cross-tier interference near FAPs. The number of users served by the FAPs is higher, when the owners' demand is lower. If the number of total users increases, RNC computes a new threshold throughput limit for the floating users in the network.

## 5. Conclusion

The placement of a femtocell has a critical effect on the performance of the wider network. This is one of the key issues to be addressed for successful deployment. The dynamic level resource-allocation scheme presented in this paper changes the environment of the network operation rapidly based of the users' congestion. Considering the minimum resources required for the FAP owners, the system ensures better utilization of available spectrums. The network can offload a certain amount of excess MBS load by diverting it to FAPs. It utilizes the unused resources and ensures a superior level of service for the roaming users. Based on the capacity optimization, it assists MBS to offload excess traffic that reduces macro-femto-interferences and escalates the network performance which is suitable for higher deployment densities of femtocell. Future research will focus on the spectrum leasing features of hybrid access femtocell network.

## Figures and Tables

**Table 1:** System parameters.

System parameters	Value/range
Number of MBS	1
Number of FAP	1–25
Number of active user	400
Number of active owners in FAP	3–5
Range of MBS	500 m
Range of FAP	20 m
MBS antenna height	30 m
FAP antenna height	1 m
User equipment height	1 m
Frequency	2 GHz
Bandwidth	10 MHz
Subcarrier spacing	15 KHz
MBS transmission power	46 dBm
Macroantenna gain	13 dBm
FAP transmission power	20 dBm
Distribution time interval	500
FAP arrival intensity	1
Random active user arrival intensity	1.5
Shadow fading std.	6 dB
White noise power density	−174 dBm/Hz
Modulation scheme	64-QAM
Number of resource blocks	50
Subcarrier per resource block	12
Resource block size	180 Khz
BER	10^−6^

**Table 2:** Owners' minimum throughput.

Set	Owners' minimum throughput
Set 1	5 Mbps
Set 2	3.5 Mbps
Set 3	2 Mbps
